# Neighborhood Disadvantage, Biological Aging, and Psychosocial Factors in Heart Failure Among Black Persons

**DOI:** 10.1093/geroni/igaf122.1305

**Published:** 2025-12-31

**Authors:** Ganga Bey, James Pike, Anthony Zannas, Qian Xiao, Amil Shah, Priya Palta

**Affiliations:** The University of North Carolina at Chapel Hill, Chapel Hill, North Carolina, United States; New York University, New York, New York, United States; The University of North Carolina at Chapel Hill, Chapel Hill, North Carolina, United States; The University of Texas Health Science Center, Houston, Texas, United States; UT Southwestern Medical Center, Dallas, Texas, United States; UNC School of Medicine, Chapel Hill, North Carolina, United States

## Abstract

Deprived living environments contribute to greater heart failure (HF) risk among non-Hispanic Black persons, who disproportionately occupy disadvantaged neighborhoods. The mechanisms for these effects are not fully explicated, partially attributable to an insufficient understanding of the individual factors that contribute additional risk or resilience to the impact of neighborhood disadvantage on health. The objective of this study was to clarify the complex pathways over which such exposures act to facilitate more targeted, effective interventions. Given the evidence for a mediating role of biological age and a moderating role of individual psychosocial characteristics in the neighborhood disadvantage-HF link, we tested a moderated mediation mechanism. Using multilevel causal moderated mediation models, we prospectively examined whether the association of neighborhood disadvantage with incident HF mediated through accelerated biological aging, captured by the GrimAge epigenetic clock, is moderated by hypothesized psychosocial risk (negative affect) and resilience (optimism) factors. Among a sample of 1,448 Black participants in the shared Jackson Heart Study-Atherosclerosis Risk in Communities cohort (mean age 64.3 years), 334 adjudicated incident hospitalized HF events occurred over a median follow-up of 18 years. In models adjusted for age and sex, the indirect (GrimAge-mediated) effect of neighborhood disadvantage was moderated by psychosocial risk such that for every standard deviation increase in negative affect the hazards of HF was 1.18 (95% confidence interval = 1.05, 1.36). No moderated mediation effect was detected for optimism. Findings support the necessity for multilevel interventions simultaneously addressing neighborhood and individual psychosocial risk in the reduction of HF among Black persons.

